# Beneficial effects of murtilla extract and madecassic acid on insulin sensitivity and endothelial function in a model of diet-induced obesity

**DOI:** 10.1038/s41598-018-36555-1

**Published:** 2019-01-24

**Authors:** Jorge Arancibia-Radich, Raquel González-Blázquez, Martín Alcalá, Miriam Martín-Ramos, Marta Viana, Silvia Arribas, Carla Delporte, María S. Fernández-Alfonso, Beatriz Somoza, Marta Gil-Ortega

**Affiliations:** 1 Laboratorio de Productos Naturales, Departamento de Química Farmacológica y Toxicológica, Facultad de Ciencias Químicas y Farmacéuticas, Universidad de Chile, PO Box 8380492, Santos Dumont 964, Santiago, Chile; 2 Departamento de Ciencias Farmacéuticas y de la Salud, Facultad de Farmacia, Universidad San Pablo-CEU, CEU Universities, 28925 Madrid, Spain; 3 Departamento de Química y Bioquímica, Facultad de Farmacia, Universidad San Pablo-CEU, CEU Universities, 28925 Madrid, Spain; 4 Departamento de Farmacología, Facultad de Farmacia, Universidad Complutense de Madrid, 28040 Madrid, Spain; 5 Instituto Pluridisciplinar, Unidad de Cartografía Cerebral, Universidad Complutense de Madrid, 28040 Madrid, Spain; 6 Departamento de Fisiología, Facultad de Medicina, Universidad Autónoma de Madrid, 28029 Madrid, Spain

## Abstract

Infusions of murtilla leaves exhibit antioxidant, analgesic, and anti-inflammatory properties. Several compounds that are structurally similar to madecassic acid (MA), a component of murtilla leaf extract (ethyl acetate extract, EAE), have been shown to inhibit protein tyrosine phosphatase 1B (PTP1P). The aim of this study was to evaluate if EAE and two compounds identified in EAE (MA and myricetin [MYR]) could have a beneficial effect on systemic and vascular insulin sensitivity and endothelial function in a model of diet-induced obesity. Experiments were performed in 5-week-old male C57BL6J mice fed with a standard (LF) or a very high-fat diet (HF) for 4 weeks and treated with EAE, MA, MYR, or the vehicle as control (C). EAE significantly inhibited PTP1B. EAE and MA, but not MYR, significantly improved systemic insulin sensitivity in HF mice and vascular relaxation to Ach in aorta segments, due to a significant increase of eNOS phosphorylation and enhanced nitric oxide availability. EAE, MA, and MYR also accounted for increased relaxant responses to insulin in HF mice, thus evidencing that the treatments significantly improved aortic insulin sensitivity. This study shows for the first time that EAE and MA could constitute interesting candidates for treating insulin resistance and endothelial dysfunction associated with obesity.

## Introduction

*Ugni molinae* Turcz, Myrtaceae (*Myrtus ugni* Mol.) is a Chilean native species commonly known as murtilla, murta or uñi that grows in the wild in southern Chile. Murtilla is a bush approximately 1.5 m high that can sometimes reach 2 m. Its fruits are eaten fresh and used in homemade jam, syrup, desserts, and liquor^1,2^. According to popular culture, murtilla has anti-inflammatory and antioxidant properties, among others^1,3^. It has been proven that the regular intake of infusions of murtilla leaves increases plasmatic antioxidant capacity, due to the high number of polyphenols, heterosides (rhamnoside, xylosides, and glucosides) of flavonols such as quercetin, myricetin, and kaempferol, in addition to genins, such as quercetin, myricetin and epicatechin^4–6^. Other researched properties of murtilla leaves are antimicrobial action^6,7^ and inhibition of enzymes involved in the control of glycaemia such as α-amylase and α-glycosidase^8^. Our previous studies carried out with wild murtilla leaves have shown that the ethyl acetate extract (EAE) and the ethanolic extract have anti-inflammatory and analgesic properties^9^. Moreover, we have contributed to the knowledge of several triterpenoid acids present in murtilla leaves, such as: oleanolic, ursolic, betulinic, alphitolic, corosolic, maslinic, asiatic, and madecassic acid^10–12^.

It is well known that obesity constitutes an independent risk factor for the development of several cardiometabolic disorders^13,14^. An association between obesity and the development of both insulin resistance and endothelial dysfunction has been described even in early stages of obesity^14–18^. Recent studies aimed at ameliorating cardiometabolic disorders associated with obesity have highlighted protein tyrosine phosphatase 1B (PTP1B) as a potential target to improve not only insulin sensitivity, but also endothelial dysfunction^19^. In this regard, selective deficiency of PTP1B in the liver significantly improves insulin sensitivity, thus leading to complete protection against obesity-induced endothelial dysfunction^20^. Moreover, several inhibitors of PTP1B, like CX08005^21^ or Norathyriol^22^, have been found to significantly improve insulin sensitivity in murine models of diet-induced obesity (DIO). Interestingly, several pentacyclic acid triterpenoids, with a similar chemical structure to madecassic acid (MA), have been observed to inhibit PTP1B activity, thus improving insulin sensitivity and stimulating glucose uptake^23^. Moreover, it has been suggested that MA could have antidiabetic effects, though further studies are required^24^.

A very recent study has suggested that murtilla fruit extract exhibits vasodilatory activity mediated by nitric oxide (NO) release in aortic rings from normal-weight Sprague Dawley rats^25^.

The hypothesis of this study is that the EAE of murtilla leaves corresponding to genotype 19-1 (EAE) has a beneficial effect on insulin resistance through the inhibition of PTP1B and on endothelial dysfunction through an increase in NO availability in a murine DIO model. In addition, since MA and myricetin (MYR) are two of the compounds identified in EAE^9^, we also wondered if the potential effects mediated by EAE could be attributed to either MA and/or MYR. Therefore, the aim of this study was to evaluate whether a 4-week treatment with either EAE, MA, or MYR modifies: i) body weight and adiposity, ii) systemic insulin sensitivity, iii) endothelial function, and iv) vascular insulin sensitivity.

## Materials and Methods

### Animals and dietary treatments

Four-week old male C57BL/6 J mice (Charles River, Spain) weighing 16–18 g were housed under controlled light (12-hour light/dark cycles from 8 am to 8 pm) and temperature (22–24 °C) conditions with standard food and water *ad libitum*. After one week of acclimation, animals with similar average body weight were divided into two groups, housed five per cage, and assigned to a control low-fat diet (LF) or to a high-fat diet (HF). Control LF (D12450B, 10 kcal % fat, 72 kcal % carbohydrates, and 18 kcal % protein; 3.76 kcal/g) and HF (D12451, 62 kcal % fat, 20 kcal % carbohydrates, and 18 kcal % protein; 5.1 kcal/g) diets were supplied by Test Diet (UK). All animals had free access to food for 4 weeks. In addition, animals were injected daily with murtilla extract (EAE, 20 mg/kg), madecassic acid (MA, 20 mg/kg), myricetin (MYR, 20 mg/kg) or saline with 1% DMSO (vehicle of the active ingredients and the extract) intraperitoneally (i.p), thus generating 8 different groups LF-C, LF-EAE, LF-MA, LF-MYR, HF-C, HF-EAE, HF-MA, and HF-MYR. On the last day, mice were weighed and euthanized by decapitation at 9 am. Blood was collected in chilled EDTA-coated polypropylene tubes. Plasma samples were stored at −80 °C for biochemical determinations. Perirenal (PR-AT), subcutaneous (SC-AT), and mesenteric adipose tissues (Mes-AT) were dissected, weighed, and their weight was normalized by tibia length. Thoracic aortas were used for both vascular function and RT-qPCR studies. The study conforms to the *Guide for the Care and Use of Laboratory Animals* published by the US National Institute of Health (NIH publication No.85–23, revised in 2011) and was approved by the Ethics Committee of the Universidad Complutense de Madrid.

### Intraperitoneal glucose tolerance test (GTT)

During the last week of dietary treatment, mice were fasted for 6 h before the glucose load (i.p. bolus of 1 g/kg at time 0). Blood glucose level was measured immediately at 0, 15, 30, 45, 60, 90, and 120 min after injection. At the indicated times, blood samples were drawn from the tail vein of conscious mice for glucose determination with an Accu-Chek Aviva glucometer (Roche Diagnostics, Germany).

### PTP1B inhibition assay *in vitro*

The protein tyrosine phosphatase 1B (PTP1P) inhibition assay was adapted from the method previously described by Na *et al*.^26^. Briefly, PTP1B activity was assessed using p-nitrophenyl phosphate (pNPP) as a substrate that is hydrolyzed by PTP1B to p-nitrophenol. Reactions were performed in 96-well plates. To each well, 12 mM pNPP and 0.1 μg of recombinant PTP1B (expressed in *E. Coli*) were added in a buffer containing 50 mM HEPES, 150 mM NaCl and 5 mM dithiothreitol (DTT) with or without the murtilla extract (EAE, 2 μg/ml) diluted in 12.5% DMSO. After 30 min of incubation at 37 °C, the reaction was stopped with 5 M NaOH. The amount of *p*-nitrophenol was estimated by measuring the absorbance at 405 nm. The non-enzymatic hydrolysis of 12 mM *p*NPP was corrected by the increase in absorbance measured at 405 nm in the absence of PTP1B.

### RNA extraction and real-time PCR (RT-qPCR)

Total RNA was isolated from the aorta using Trizol Reagent (Invitrogen, Spain). The samples were processed using an RNeasy Mini Kit (Qiagen, Spain). The concentration, purity, and integrity of RNA was assessed with NanoVue (GE Healthcare, Germany). Reverse transcription was performed on 500 ng of RNA with an iScript cDNA synthesis kit (BioRad, CA) using random hexamer primers. Optimal annealing temperature and amplicon sizes were checked for each pair of primers. RT-qPCR analyses were performed in a CFX96 Instrument (BioRad, CA). The primer sequence for the PTP1B gene, *Ptpn1* (accession number NM_011201.3) was 5′-GGAGACCTGTGGGGATGA-3′ (forward) and 5′-TCAGTGTCTGGACTCATGCTG-3′ (reverse) and for the housekeeping gene, Tbp (accession number NM_013684.3), 5′-ACCCTTCACCAATGACTCCTATG-3′ (forward) and 5′-TGACTGCAGCAAATCGCTTGG-3′ (reverse). A total of 6.25 ng of cDNA from 4 samples of each group were run in triplicate and the mRNA levels were determined using intron-skipping primers, TBP as a housekeeping gene, and SYBR Green Master Mix (Applied Biosystems, CA).

### Vascular reactivity in isolated aortic arteries cleaned of PVAT

Vascular reactivity experiments were performed as previously described^27^. Briefly, aortic rings of 2–3 mm in length were given an optimal resting tension of 1.5 g, which was readjusted every 15 min for a 40-min equilibration period. Before starting the experiment, rings were contracted with 75 mM KCl to assess their contractility. Contractile responses were assessed by the incubation of aortic segments with phenylephrine (Phe, 10^−8^ to 10^−6^M). Endothelial integrity was analyzed by addition of acetylcholine (Ach, 10^−9^ to 10^−4^M) to segments pre-contracted with Phe (10^−7^ to 10^−6^M to reach an equivalent tone between groups). The nitric oxide synthase inhibitor, NG-nitro-L-arginine methyl ester (L-NAME, 10^−4^M) was pre-incubated for 20 min before adding Phe (10^−7^ to 10^−6^M). NO contribution was assessed by calculating the percentage of inhibition of Ach-induced relaxations by L-NAME. Relaxant responses to insulin were determined by addition of insulin (10^−10^–10^−5^M) to segments pre-contracted with Phe (10^−7^ to 10^−6^M). Endothelium-independent relaxation was analyzed by addition of sodium nitroprusside (SNP, 10^−12^–10^−5^M) to segments pre-contracted with Phe (10^−7^ to 10^−6^M).

### Cell culture experiments

Bovine aortic endothelial cells (BAECs) were obtained from the European collection (ECACC; Sigma-Aldrich, Spain). BAECs were cultured in complete medium (Dulbecco’s modified Eagle’s medium [DMEM] with 10% fetal bovine serum [FBS], L-glutamine [2·10^−3^M], penicillin [100 U/mL], and streptomycin [100 μ/mL] from LONZA, USA) and maintained at 37 °C in a humidified atmosphere consisting of 5% CO_2_ and 95% air. All the experiments were performed in confluent cells (90%), between passages 3 and 6 and cultured in DMEM and 0.1% of bovine serum albumin (BSA), thus being deprived of serum for 20 h. Under these conditions, cells were treated with insulin (5·10^−7^M) for 10 min, with EAE (40 μg/mL) or MA (5·10^−4^M) for 15 min, or nothing as a control. After these incubations, cells were briefly washed with ice-cold PBS and were then scraped in lysis buffer containing 0.42 mM NaCl, 1 mM Na_4_P_2_O_7_, 1 mM dithiothreitol, 20 mM HEPES, 20 mM NaF, 1 mM Na_3_VO_4_, 1 mM EDTA, 1 mM EGTA, 20% glycerol, 2 mM phenylmethylsulfonyl fluoride, 1 µl/ml leupeptin, 1 µl/ml aprotinin, and 0.5 µl/ml Tosyl-L-lysyl-chloromethane hydrochloride. Cells lysates were centrifuged at 17.000xG for 20 min at 4 °C and the supernatants were used for Western blot studies.

### Confocal microscopy experiments

BAECs were directly plated on 24 mm^2^ glass cover slips and subsequently used for evaluating endogenous NO production. Cells, previously deprived of serum and treated with insulin, EAE, or MA, as previously described (see cell culture experiments), were incubated with a fluorescent NO indicator 4,5-diaminofluorescein diacetate (DAF-2DA, 10^−5^M; Sigma-Aldrich, USA) for 30 min. Thereafter, cells were washed with cold PBS and fixed in paraformaldehyde (PFA) at 4%. Nuclei were stained by incubation with 4′,6-diamino-2-phenylindole (DAPI, 1:500 from stock 5 mg/ml; Molecular Probes, USA) for 15 min at room temperature in the dark and cells were washed with PBS. The cover slips with BAECs were mounted in a glycerol/PBS solution (Citifluor, VWR International, Spain) and analyzed by confocal microscopy as previously described^28^. From each culture, a minimum of 3 randomly selected images were captured with a LEICA SP5 confocal microscope (Leica Microsystems) using the 488 nm/530 nm (DAF-2DA, NO) and 405 nm/410–475 nm (DAPI, nuclei) filters with a x20 objective.

### Western blot analyses

p-AKt/AKt and p-eNOS/eNOS expression was determined in cells treated with insulin, EAE, or MA by Western blot, as previously described^29^. Briefly, 30 µg protein samples were separated by 10% SDS-PAGE gels. Primary antibodies against e-NOS (BD Transduction Laboratories, UK; 1:1000 final dilution), p-eNOS (Ser-1177) (Cell Signalling Technology, USA; 1:500 final dilution), AKt (Cell Signalling Technology, USA; 1:500 final dilution) and p-AKt (Ser-473) (Cell Signalling Technology, USA; 1:1000 final dilution) were applied overnight at 4 °C. After washing, appropriate secondary antibodies (anti-rabbit IgG-peroxidase conjugated) were applied for 1 h. Blots were washed, incubated in commercial enhanced chemiluminescence reagents (ECL, Amersham Bioscience, UK) and analyzed with ChemiDoc (BIO-RAD, Spain). To prove equal loadings of samples, blots were re-incubated with β-actin antibody (Sigma-Aldrich, Spain). Expression values of p-AKt and peNOS were normalized with AKt and eNOS, respectively.

### Chemicals

Ach was dissolved in saline, Phe and SNP in 0.01% ascorbic acid/saline and L-NAME and insulin in distilled water (Sigma-Aldrich, USA). Madecassic acid, myricetin (Santa Cruz Biotechnology) and murtilla extract were dissolved in DMSO.

### Data analyses

All values are given as mean ± SEM and *n* denotes the number of animals used in each experiment. Statistical significance was analyzed using one-way or two-way analysis of variance (ANOVA) followed by Bonferroni or Newman-Keuls post-hoc test for comparison between groups. A value of *p* < 0.05 was considered statistically significant. In vascular reactivity experiments, contractions are expressed as the percentage of contraction elicited by 75 mM KCl. Relaxations are expressed as the percentage of previous Phe contraction. The maximum response (E_max_ values) was calculated by nonlinear regression analyses of each individual concentration-response curve. The area under the curves (AUC) were calculated from the individual concentration-response curve plots (GraphPad Software, USA).

## Results

### Body weight, adiposity, and glucose tolerance

After 4 weeks of diet, HF-C mice exhibited a significantly higher body weight than the control group (LF-C:22.8 ± 0.6 g *vs*. HF-C:24.3 ± 0.7, p < 0.05). Neither treatment with EAE nor with MYR modified body weight after the HF diet (Table 1). However, the treatment with MA significantly reduced the body weight of HF mice to the level of the LF-C group (HF-MA:22.3 ± 0.8; p < 0.05 compared to HF-C). These changes were not attributable to differences in food intake, since it was similar in all groups (Supplementary Figure 1). No effects of treatment were observed on the body weight of LF groups.

**Table 1:** Effect of dietary treatment on body weight and adiposity.

	LF-C	LF-EAE	LF-MA	LF-MYR	HF-C	HF-EAE	HF-MA	HF-MYR
Body weight (g)	22.8 ± 0.6	22.3 ± 0.5	23.0 ± 0.6	21.7 ± 0.9	24.3 ± 0.7^*^	24.5 ± 1.2	22.8 ± 0.8	23.5 ± 0.6
PR-AT (mg/mm)	3.9 ± 0.5	3.6 ± 0.3	2.8 ± 0.2	2.9 ± 0.3	6.8 ± 1.1^*^	6.5 ± 1.3	6.5 ± 0.7	4.8 ± 1.1
SC-AT (mg/mm)	15.3 ± 1.6	15.1 ± 0.7	12.6 ± 0.7	17.0 ± 1.6	20.1 ± 1.9^*^	21.2 ± 2.7	17.1 ± 1.3	14.6 ± 2.1^#^
Mes-AT (mg/mm)	3.8 ± 0.6	4.1 ± 0.6	3.0 ± 0.2	2.4 ± 0.2	4.0 ± 0.9	4.2 ± 0.9	4.5 ± 0.3	3.1 ± 0.3
Tibial length (mm)	22.3 ± 0.2	22.3 ± 0.2	22.5 ± 0.3	22.0 ± 0.3	22.4 ± 0.2	22.4 ± 0.4	22.2 ± 0.4	22.1 ± 0.4

Adipose tissue weights were normalized using the tibial length, which was not modified by the high-fat diet. Data on body and adipose tissues are expressed as mean ± SEM of 5 determinations per group. ^*^p < 0.05 compared with control group LF; ^#^p < 0.05 compared with control group HF.

The amount of both PR-AT and SC-AT was higher in HF-C animals than in LF-C, but no differences were detected in Mes-AT (Table 1). Treatment with EAE or MA did not modify the amount of SC-AT and PR-AT in HF or LF animals. However, MYR significantly reduced the amount of SC-AT in HF mice (p < 0.05). Nevertheless, treatment did not affect animal growth as assessed by tibial length (Table 1).

**Figure 1:**
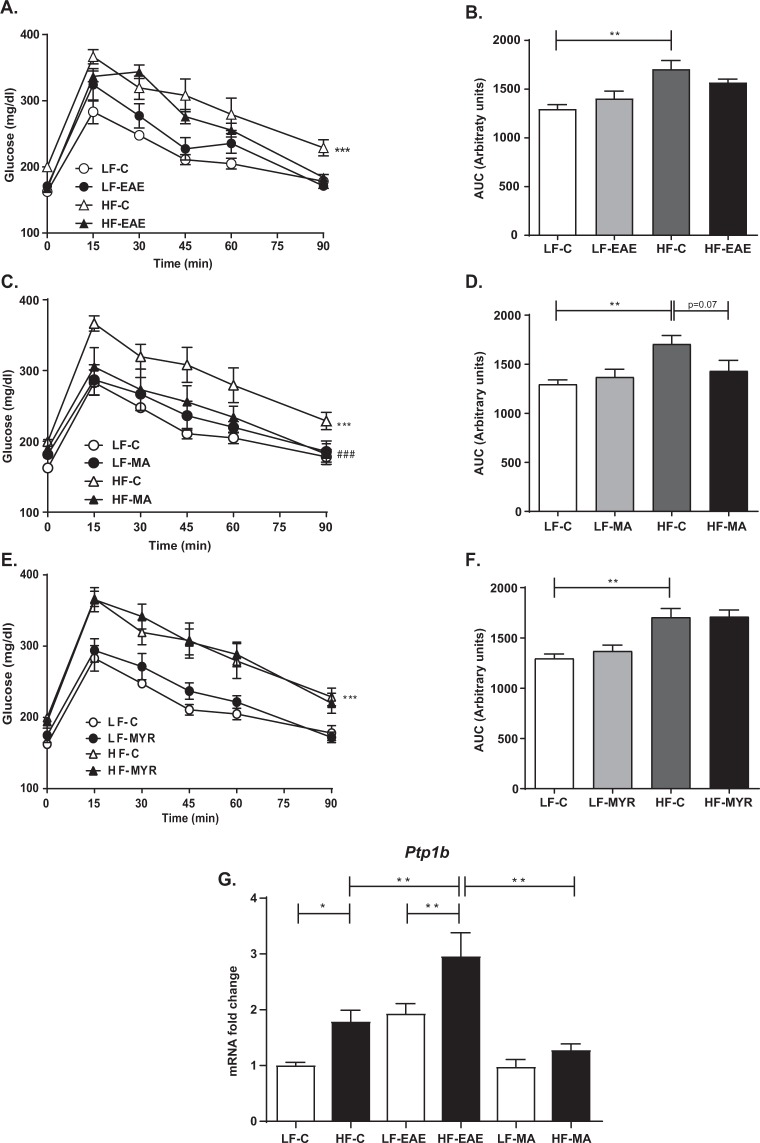
GTT (**A**,**C**,**E**) and AUC during the GTT (**B**,**D**,**F**) in LF and HF mice treated or not with EAE, MA, or MYR, respectively. (**G**) Diagram bars show PTP1B mRNA expression in aortas from LF and HF mice treated or not with EAE or MA. Data are expressed as mean ± SEM of 5 determinations per group. ^*^p < 0.05, ^**^p < 0.01, and ^***^p < 0.001 compared with LF-C (**A**,**C**,**E**) or the matched group (**B**,**D**,**E**,**G**). ^###^p < 0.001 compared with HF-C.

As shown in Fig. 1, the GTT area under the curve (AUC) was significantly higher in HF-C animals than in the LF-C group, which suggests that there was significant glucose intolerance in HF-C mice (Fig. 1). The treatment with MA (Fig. 1C) significantly improved the glucose tolerance in HF mice. Indeed, HF-MA mice even exhibited similar GTT AUC to LF-C mice (Fig. 1D). The treatment with EAE induced a faster reduction in glucose levels in HF than in HF-C, as shown in glucose levels 90 min after glucose overload (HF-C: 228.8 ± 12.3 *vs*. HF-EAE: 181.8 ± 14.7, p < 0.05) (Fig. 1A). Nevertheless, the effect was not strong enough to reflect differences in the AUC (Fig. 1B). The treatment with MYR did not modify glucose tolerance (Fig. 1E,F).

### Inhibition of PTP1B by EAE *in vitro* and PTP1B expression in the murine aorta

EAE, used at a concentration of 2 μg/mL, significantly inhibited the PTP1B, with 97.2 ± 0.9% percentage of inhibition. This result suggests a role for EAE in the regulation of both insulin sensitivity and glucose uptake.

In addition, since insulin sensitivity was significantly impaired in HF mice and relaxant responses to insulin were compromised in aortic segments from HF mice, we also evaluated PTP1B mRNA expression in aorta. As expected, PTB1B expression was significantly enhanced in aorta segments by the HF diet (p < 0.05), except in HF mice treated with MA, which exhibited similar values to the LF-C group (Fig. 1G).

### Treatment with EAE or MA improved endothelial function in HF mice

**Figure 2:**
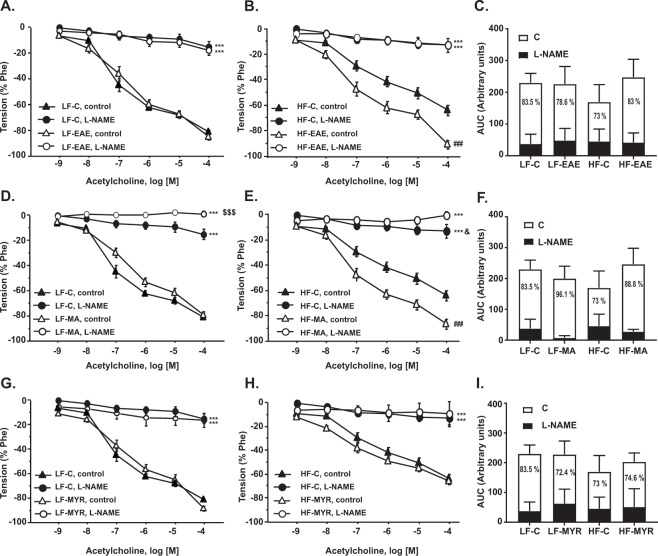
Cumulative concentration-response curves to acetylcholine (10^−9^–10^−4^M) in aortic segments pre-incubated or not with L-NAME (10^−4^M) from LF mice treated or not with EAE (**A**), MA (**D**), or MYR (**G**) and HF mice treated or not with EAE (**B**), MA (**E**), or MYR (**H**). Bar diagrams showing AUC from cumulative concentration-response curves to acetylcholine (10^−9^–10^−4^M) in aortic segments from mice treated or not with EAE (**C**), MA (**F**), or MYR (**I**). The percentage of inhibition elicited by L-NAME and shown in white bars indirectly reflects the NO contribution to ACh-induced relaxant responses. Data are expressed as mean ± SEM of 5 determinations per group. ^***^p < 0.001 compared with the corresponding control group; ^###^p < 0.001 compared with the HF-C group; ^$^p < 0.05, ^$$$^p < 0.001 compared with LF-C L-NAME; ^&^p < 0.05 compared with HF-C L-NAME.

Endothelial function was evaluated by analyzing endothelium-dependent relaxations to Ach (10^−9^ to 10^−4^ M) in aortic segments pre-constricted with Phe. Relaxant responses to Ach were significantly lower in aortic segments from HF-C mice than those from LF-C, which evidences an endothelial dysfunction in aortas from HF mice (E_max_ LF-C:81.5 ± 1.5 *vs*. E_max_ HF-C:64.1 ± 4.2, p < 0.001, Fig. 2 and Table 2). In HF mice, treatment with EAE (Fig. 2B) and MA (Fig. 2E) restored Ach-induced relaxation, which showed similar values to the LF-C group (Fig. 2A,D, respectively). However, MYR did not modify the response to Ach in LF or HF mice (Fig. 2G,H).

**Table 2:** Emax and AUC values from cumulative concentration curves to Ach (10-9-10-4 M) in the presence or not of the NO synthase inhibitor (L-NAME).

	LF-C	LF-EAE	LF-MA	LF-MYR	HF-C	HF-EAE	HF-MA	HF-MYR
Emax ACh	81.5 ± 1.5	85.0 ± 2.4	79.6 ± 3.0	89.1 ± 1.8^***^	64.1 ± 4.2^***^	91.0 ± 3.2^###^	86.5 ± 3.6^###^	64.4 ± 3.3
Emax (L-NAME)	16.5 ± 3.2	18.4 ± 3.8	0.5 ± 2.5^*^	17.1 ± 5.8	13.0 ± 5.4	12.5 ± 2.1	0.5 ± 1.5^###^	9.4 ± 10.5
AUC ACh	183.3 ± 18.5	200.4 ± 10.3	206.9 ± 18.6	181.3 ± 10.3	147.4 ± 16.4	229.5 ± 25.3^##^	273.0 ± 17.8^###^	202.7 ± 11.7^#^
AUC (L-NAME)	41.5 ± 10.5	52.5 ± 17.1	15.5 ± 7.8^**^	62.8 ± 21.6	45.7 ± 12.8	33.9 ± 12.9	28.2 ± 3.2	64.5 ± 31.1

Data are expressed as mean ± SEM of 5 determinations per group ^*^p < 0.05, ^**^p < 0.01, and ^***^p < 0.001 compared with LF-C; ^#^p < 0.05, ^##^p < 0.01, and ^###^p < 0.001 compared with HF-C.

To assess whether the treatment with EAE or MA increased NO availability, aortic rings were pre-incubated with L-NAME (10^−4^M). In the presence of L-NAME, Ach-induced relaxations were significantly reduced in both LF-C and HF-C groups (E_max_ LF-C:16.5 ± 3.2 *vs*. E_max_ HF-C:13.0 ± 5.4, Fig. 2). Nevertheless, the percentage of inhibition elicited by L-NAME (calculated from the difference between the AUC in the presence and absence of L-NAME that indirectly indicates ACh-induced NO release) was lower in the HF-C group (73%) than in the LF-C group (83.5%) (Fig. 2C, see NO contribution in white). In addition, the treatment with both EAE and MA, but not with MYR, significantly increased Ach-induced NO release to reach normal values (% of inhibition in HF-EAE: 83%, Fig. 2C; % of inhibition in HF-MA: 88.8%, Fig. 2F; % of inhibition in HF-MYR: 74.6%, Fig. 2I).

Moreover, smooth muscle sensitivity to NO, assessed with concentration-response curves to a NO donor SNP (10^−12^–10^−5^M) was not modified by the HF diet or by EAE or MA. However, HF mice treated with MYR exhibited a significant reduction of relaxation elicited by SNP (Supplementary Figure 2).

**Figure 3:**
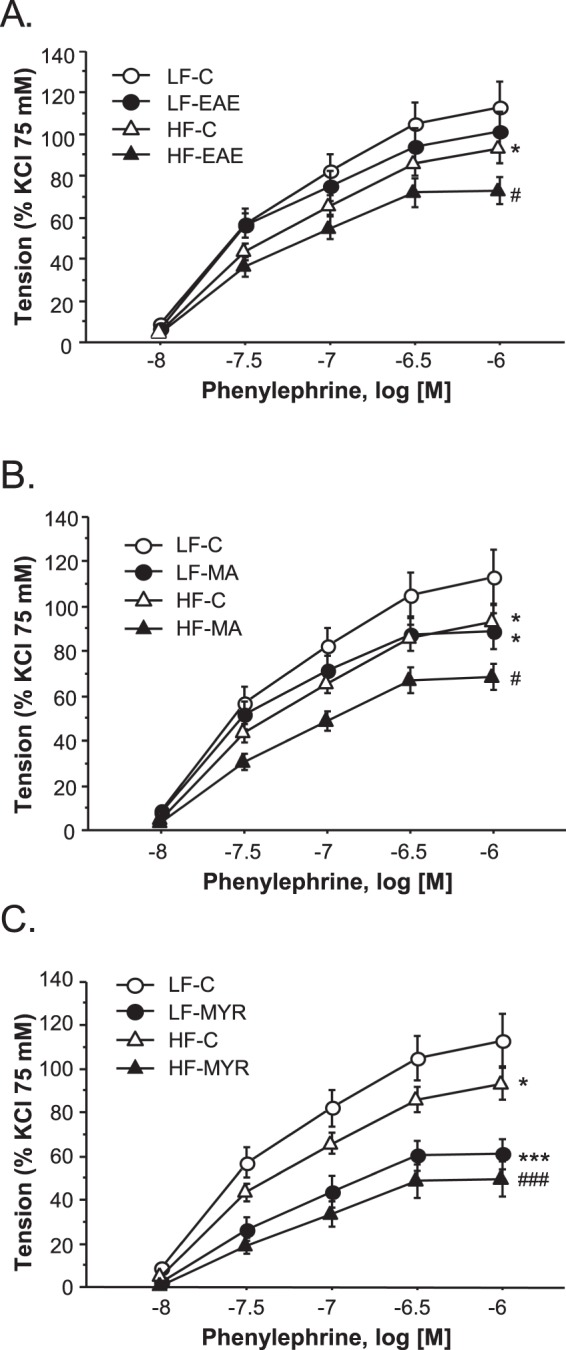
Cumulative concentration-response curves to phenylephrine (Phe, 10^−8^–10^−6^M) in aortic segments from LF and HF animals treated or not with EAE (**A**), MA (**B**), or MYR (**C**). Data are expressed as mean ± SEM of 5 determinations per group. ^*^p < 0.05 and ^***^p < 0.001 compared with the LF-C group; ^#^p < 0.05 and ^###^p < 0.001 compared with the HF-C group.

To assess the effects of treatments on vascular contractility, concentration-response curves to Phe (10^−8^M–10^−6^M) were performed in aortic segments. Contractile responses to Phe were significantly smaller in the HF-C group than in the LF-C group (p < 0.05). In addition, treatment with EAE, MA, or MYR reduced Phe-induced contractions in HF animals (p < 0.05, p < 0.05, and p < 0.001, respectively compared to the HF-C group, Fig. 3).

### Treatment with EAE, MYR, or MA enhanced relaxant responses to insulin in HF mice

**Figure 4:**
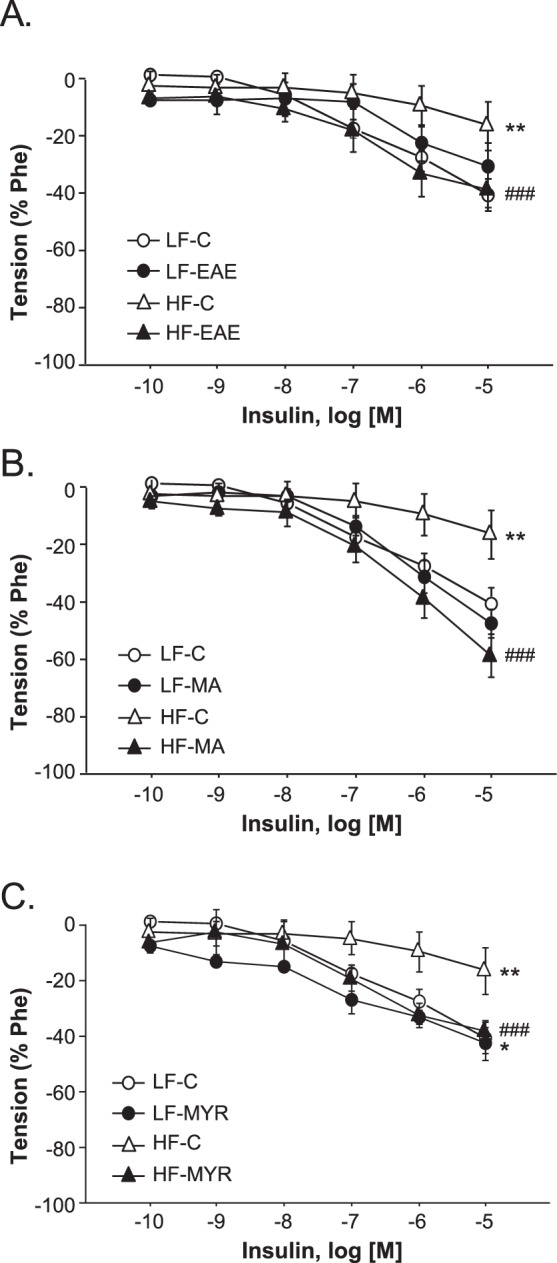
Cumulative concentration-response curves to insulin (10^−10^–10^−5^M) in aortic segments pre-constricted with Phe from LF and HF animals treated or not with EAE (**A**), MA (**B**), or MYR (**C**). Data are expressed as mean ± SEM of 5 determinations per group. ^*^p < 0.05 and ^**^p < 0.01 compared with the LF-C group; ^###^p < 0.001 compared with the HF-C group.

To analyze the effect of treatments on aortic insulin sensitivity, we performed concentration-responses curves to insulin (10^−10^M-10^−5^M) in vessels pre-constricted with Phe. As shown in Fig. 4, relaxant responses to insulin were significantly compromised in aortic segments from HF-C mice (p < 0.05) compared to the LF-C group. However, when treated with EAE, MA, or MYR, aortic segments from HF mice exhibited similar relaxant responses to insulin to the LF-C group (Fig. 4A–C).

### Both EAE and MA increased NO availability in BAECs through AKT and eNOS phosphorylation

**Figure 5:**
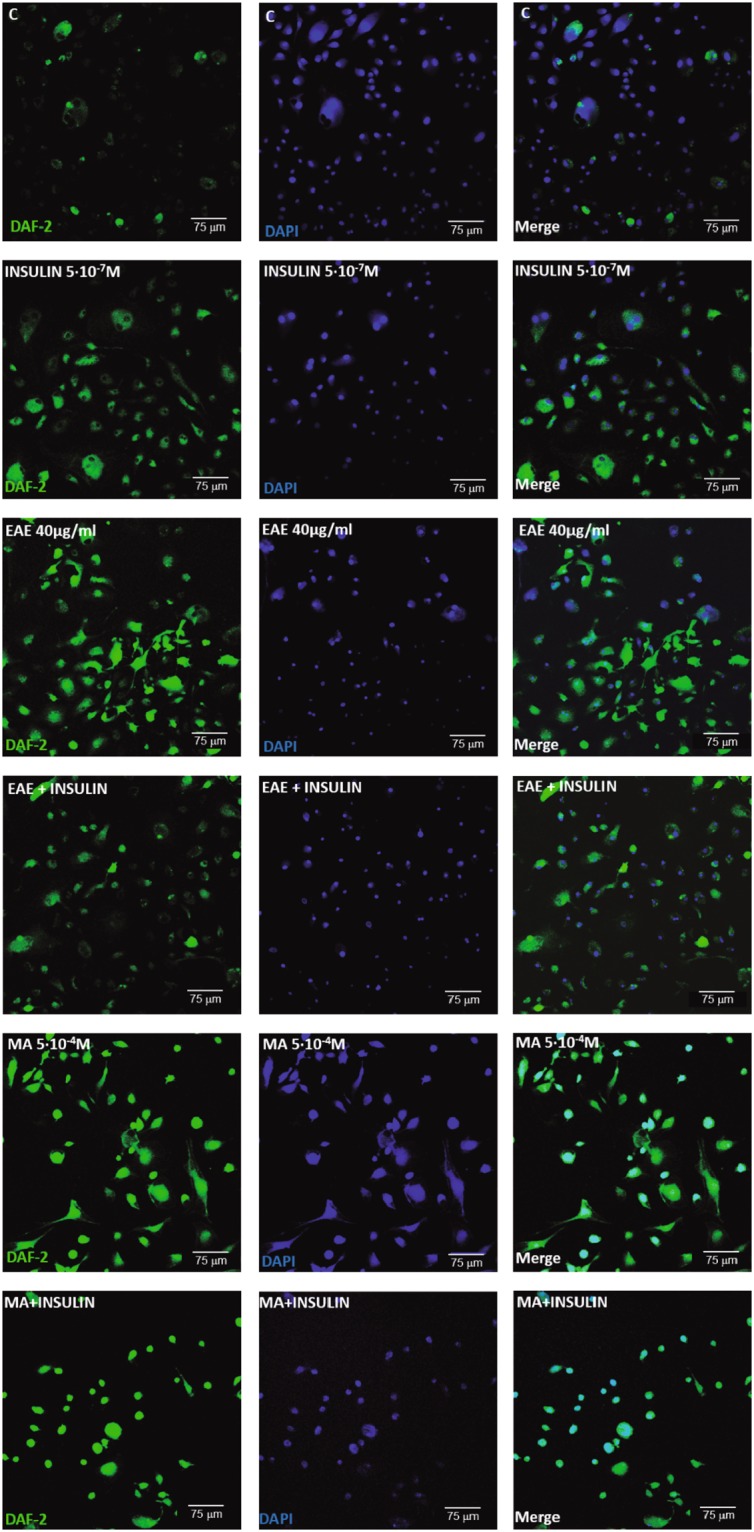
Confocal projections showing *in situ* nitric oxide (NO) availability determined with DAF-2 DA (10^−5^M, in green), nuclei staining with DAPI (in blue) and merged images in BAECs treated with EAE (40 µg/ml) or MA (5^*^10^−4^M) with or without insulin (5^*^10^−7^M) or nothing as a control.

Since both EAE and MA, but not MYR, significantly enhanced NO availability in the murine aorta (Fig. 2), we next analyzed by confocal microscopy whether EAE or MA might directly induce NO production in BAECs treated with EAE or MA. Insulin was used as a positive control. As expected, fluorescence intensity and thus NO availability was significantly enhanced by the treatment with EAE, MA, or insulin compared with the controls (Fig. 5).

**Figure 6:**
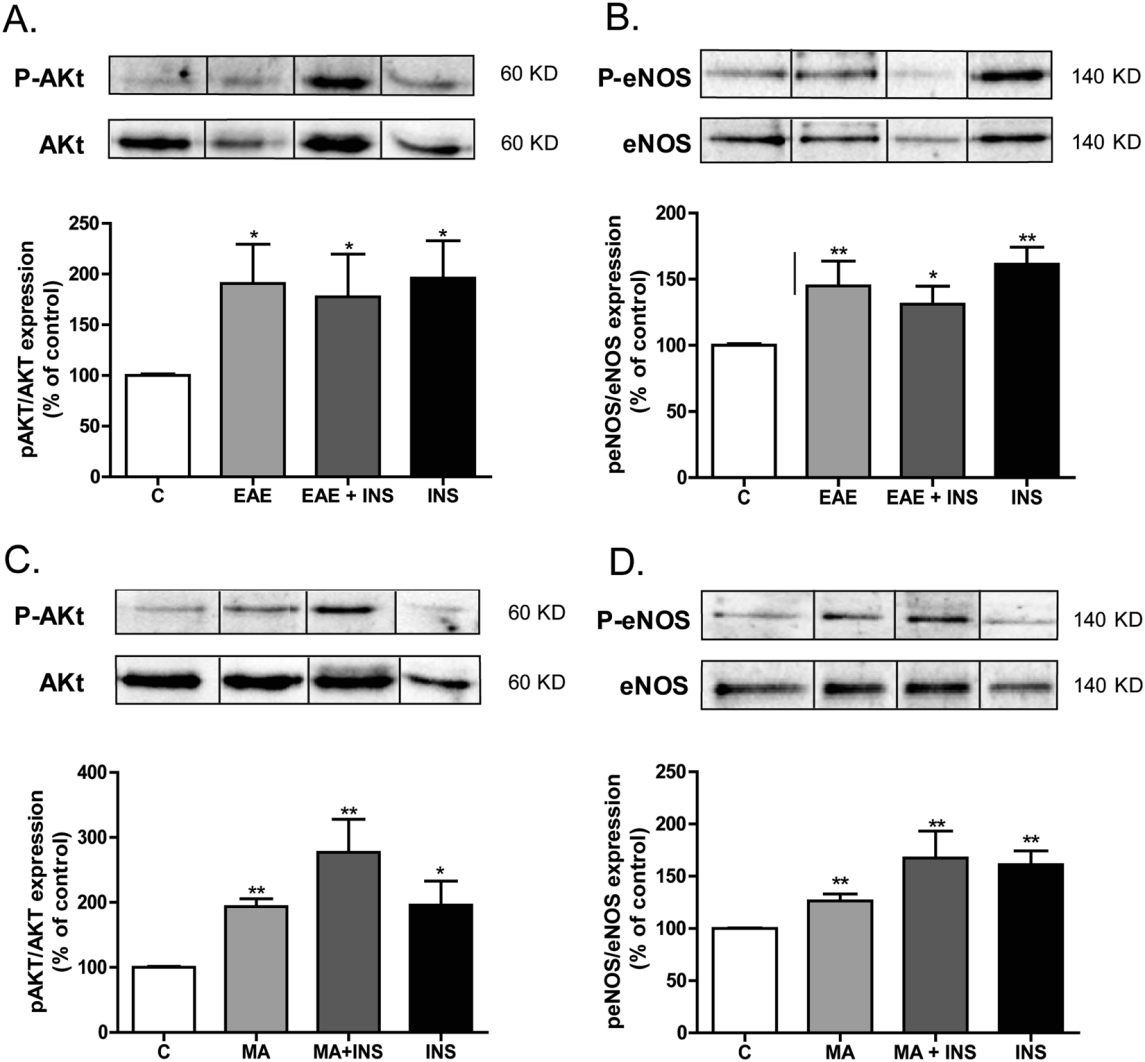
Representative immunoblots of p-Akt and Akt expression in BAECs treated or not with EAE (40 µg/ml) (**A**), or MA (5^*^10^−4^M) (**C**), insulin (5^*^10^−7^M), or both. Diagram bars show the result of densitometric analysis of p-Akt immunoblots expressed as a percentage of p-Akt/Akt in the C group. Representative immunoblots of p-eNOS and eNOS expression in BAECs treated or not with EAE (40 µg/ml) (**B**), or MA (5^*^10^−4^M) (**D**), insulin (5^*^10^−7^M), or both. Blots have been cropped from different parts of the same gel. Full-length blots are presented in Supplementary Figure 3. Diagram bars show the result of densitometric analysis of p-eNOS immunoblots expressed as a percentage of p-eNOS/eNOS in the C group. Data are expressed as mean ± SEM of ≥ 3 determinations per group. ^*^p < 0.05 and ^**^p < 0.01 compared with C cells.

In addition, Western blot studies revealed that treatment with EAE or MA significantly increased AKt phosphorylation in BAECs, as shown in Fig. 6A (p < 0.01) and 6C (p < 0.05), respectively, compared to the control group and similarly to BAECs treated with insulin. Moreover, eNOS phosphorylation (p-eNOS-Ser^1177^) was also enhanced by both EAE and MA compared with the controls (Fig. 6B [p < 0.05] and 6D [p < 0.01], respectively), and reached similar values to those mediated by insulin. Nevertheless, combined treatment with EAE or MA and insulin did not enhance the effect observed with single treatments (Fig. 6B,D).

## Discussion and Conclusions

In this study, we demonstrate for the first time that murtilla extract (EAE) inhibits PTP1B activity and significantly improves i) systemic glucose tolerance, ii) endothelial function, and iii) vascular insulin sensitivity in a DIO model in mice. The amelioration of vascular function induced by treatment with EAE in HF mice was found to be due to an increase in NO bioavailability. Moreover, we show that benefits of EAE in obesity could be directly mediated by MA, at least in part, since MA is one of the compounds identified in EAE and it induces similar effects to EAE.

Several studies performed in different models of DIO in mice^18,30,31^, rats^32,33^, and even observational studies in humans^14,34,35^ have largely demonstrated that cardiometabolic disorders emerge during the onset of obesity. In fact, it has been described that several disorders like insulin resistance or endothelial dysfunction might appear even at early stages in the development of obesity^14,18,30–35^. Because insulin-sensitizing drugs are limited, it is a priority to identify new potential candidates. In this regard, recent studies aimed at ameliorating cardiometabolic disorders associated with obesity have highlighted PTP1B as a potential target to improve not only insulin sensitivity, but also endothelial dysfunction^19^. Indeed, several inhibitors of PTP1B, like CX08005^21^ or Norathyriol^22^, have been found to significantly improve insulin sensitivity in murine models of DIO. Here, we have demonstrated the ability of EAE to inhibit PTP1B activity. In addition, glucose tolerance was improved in HF animals treated with EAE, though this effect was not as strong as the effect induced by MA. Interestingly, several compounds with a similar chemical structure to MA have been found to inhibit PTP1B^23^. A potential antidiabetic effect of MA^24^ has also been described. As MA is indeed one of the components of EAE, we could speculate that MA could be responsible, at least in part, for the effects observed with EAE. The improvement of glucose tolerance induced by treatment with EAE and MA was not observed when animals were treated with other compounds present in EAE and with a different chemical structure than MA, for example, the flavonol MYR. Therefore, we could discard a role for MYR in the amelioration of glucose tolerance induced by EAE in DIO mice.

Agouni *et al*. have suggested that PTP1B deletion improves insulin sensitivity, which directly accounts for the protection of endothelial function in obesity^20^. In this context, the second aim of this work was to assess if MA or EAE could improve both endothelial function and aortic insulin sensitivity in our model of DIO in mice. We found a significant improvement of relaxation to Ach in aortic segments from HF mice when treated with EAE or MA compared with the controls, which demonstrates for the first time a beneficial/protective effect of both EAE and MA in preventing the development of endothelial dysfunction associated with obesity. These results are in accordance with a study performed by Jofre *et al*. that described a vasodilatory effect of an extract of murtilla fruit in aorta from SD rats and suggested that this effect might be mediated by NO^25^. Similarly, within this study we demonstrate that the increase in Ach-induced relaxation in obese mice induced by both EAE and MA was also due to increased NO availability through enhancement of eNOS phosphorylation. It should be noted that several other compounds present in EAE like quercetin, epicatechin or MYR have shown to be able to induce vascular responses. Epicatechin and quercetin might exert vascular relaxation under physiologic circumstances^36,37^. However, a very recent study demonstrated that in overweight/obese adults, the acute intake of quercetin cannot improve endothelial function^38^. Controversial information is available regarding the vascular effects of myricetin, since it has been found to elicit both contractile^39^ and relaxant responses^40^, at low or high concentrations, respectively. In our model, MYR did not enhance Ach-induced relaxation in aortas from HF mice as both MA and EAE did. Therefore, we cannot discard a potential minor contribution of components other than MA contained in EAE on the beneficial effects elicited by the treatment with EAE on both insulin sensitivity and endothelial function in HF mice. However, a central role for MA seems to be clear. Intriguingly, since endothelial function was significantly impaired in HF-C mice compared to LF-C mice due to reduced NO contribution, we would have expected to observe an increased contractile response to NA in HF-C mice. Nevertheless, we did observe significantly fewer Phe-induced contractions in HF-C mice than in LF-C animals. These results could suggest that the increase of sympathetic activity that has been shown to occur in obesity^41^ could possibly account for desensitization of α1-receptors, though further studies should be performed to clarify this question.

Finally, we also detected a significant improvement in insulin relaxations in aortas from HF mice treated with EAE, MA, or MYR compared to HF-C mice, which reveals that treatment with EAE, MA or MYR improved aortic insulin sensitivity. Intriguingly, and in contrast to MA and EAE that ameliorated systemic glucose tolerance in HF mice, MYR did not modify systemic glucose tolerance in our model of DIO. Nevertheless, Liu *et al*. demonstrated that MYR could ameliorate insulin resistance in rats fed with a high fructose diet^42^. Therefore, although these data might suggest a potential beneficial effect of MYR mainly on aortic insulin resistance, a key role for MYR in EAE-induced improvement of both systemic glucose tolerance and endothelial function could be discarded.

In conclusion, this study shows for the first time that EAE and one of its components, madecassic acid, could constitute potentially interesting candidates for the treatment of both insulin resistance and endothelial dysfunction associated with obesity.

## Electronic supplementary material

Supplementary Figures

## References

[1] Munoz, S., M. Barrera, M. & E. Meza P., J. El uso medicinal y alimenticio de plantas nativas y naturalizadas en Chile. *Museo de Historia Natural*, *Santiago*, *Chile*. **Publicación ocasional 33** (1981).

[2] Wilhelm de Mosbach, E. In Botánica indígena de Chile. *Ed*. *Andrés Bello*, *Santiago*, *Chile* (1999).

[3] Montenegro, G. Chile nuestra flora útil. Guía de plantas de uso apícola, en medicina folclórica artesanal y ornamental. *Universidad Católica de Chile*, 2 (2000).

[4] Avello MPE (2005). Actividad antioxidante de infusos de Ugni molinae Turcz (murtilla). Bol Latinoam Caribe Plant Med Aromat.

[5] Rubilar M (2006). Murta leaves (Ugni molinae Turcz) as a source of antioxidant polyphenols. Journal of agricultural and food chemistry.

[6] Shene C (2012). *In vitro* activity on human gut bacteria of murta leaf extracts (Ugni molinae turcz.), a native plant from Southern Chile. Journal of food science.

[7] Avello M, V. R., Mondaca, M., Ordoñez, J., Bittner, M. & Becerra, J. Actividad de Ugni molinae frente a microorganismos de importancia clínica. *Bol*. *Latinoam*. *Caribe Plant Med Aromat* **2** (2009).

[8] Rubilar M (2011). Extracts of Maqui (Aristotelia chilensis) and Murta (Ugni molinae Turcz.): sources of antioxidant compounds and alpha-Glucosidase/alpha-Amylase inhibitors. Journal of agricultural and food chemistry.

[9] Arancibia-Radich J (2016). Comparative study of anti-inflammatory activity and qualitative-quantitative composition of triterpenoids from ten genotypes of Ugni molinae. Bol Latinoam Caribe Plant Med Aromat.

[10] Aguirre MC (2006). Topical anti-inflammatory activity of 2alpha-hydroxy pentacyclic triterpene acids from the leaves of Ugni molinae. Bioorganic & medicinal chemistry.

[11] Delporte C (2007). Analgesic activity of Ugni molinae (murtilla) in mice models of acute pain. Journal of ethnopharmacology.

[12] Goity, L. E. *et al*. An HPLC-UV and HPLC-ESI-MS based method for identification of anti-inflammatory triterpenoids from the extracts of Ugni molinae. *Bol Latinoam Caribe Plant Med Aromat* **12****(****1****)** (2013).

[13] Manson JE (1995). Body weight and mortality among women. N Engl J Med.

[14] Steinberg HO (1996). Obesity/insulin resistance is associated with endothelial dysfunction. Implications for the syndrome of insulin resistance. The Journal of clinical investigation.

[15] Duncan ER (2008). Effect of endothelium-specific insulin resistance on endothelial function *in vivo*. Diabetes.

[16] Duncan ER (2007). Accelerated endothelial dysfunction in mild prediabetic insulin resistance: the early role of reactive oxygen species. American journal of physiology. Endocrinology and metabolism.

[17] Williams IL (2006). Effect of fat distribution on endothelial-dependent and endothelial-independent vasodilatation in healthy humans. Diabetes, obesity & metabolism.

[18] Gil-Ortega M (2014). Imbalance between Pro and Anti-Oxidant Mechanisms in Perivascular Adipose Tissue Aggravates Long-Term High-Fat Diet-Derived Endothelial Dysfunction. PloS one.

[19] Thiebaut PA, Besnier M, Gomez E, Richard V (2016). Role of protein tyrosine phosphatase 1B in cardiovascular diseases. Journal of molecular and cellular cardiology.

[20] Agouni A (2014). Hepatic protein tyrosine phosphatase 1B (PTP1B) deficiency protects against obesity-induced endothelial dysfunction. Biochemical pharmacology.

[21] Zhang X (2016). A novel protein tyrosine phosphatase 1B inhibitor with therapeutic potential for insulin resistance. British journal of pharmacology.

[22] Ding H (2014). Norathyriol reverses obesity- and high-fat-diet-induced insulin resistance in mice through inhibition of PTP1B. Diabetologia.

[23] Ramirez-Espinosa JJ (2011). Antidiabetic activity of some pentacyclic acid triterpenoids, role of PTP-1B: *in vitro*, in silico, and *in vivo* approaches. European journal of medicinal chemistry.

[24] Hsu YM, Hung YC, Hu L, Lee YJ, Yin MC (2015). Anti-Diabetic Effects of Madecassic Acid and Rotundic Acid. Nutrients.

[25] Jofre I (2016). Antioxidant and Vasodilator Activity of Ugni molinae Turcz. (Murtilla) and Its Modulatory Mechanism in Hypotensive Response. Oxidative medicine and cellular longevity.

[26] Na M (2010). Protein tyrosine phosphatase 1B inhibitory activity of 24-norursane triterpenes isolated from Weigela subsessilis. Phytotherapy research: PTR.

[27] Steireif, C. *et al*. Dissecting the genetic predisposition to albuminuria and endothelial dysfunction in a genetic rat model. *Journal of hypertension* 31, 2203–2212; discussion 2212 (2013). 10.1097/HJH.0b013e3283642384 23868088

[28] Gonzalez JM (2008). Hypertension increases middle cerebral artery resting tone in spontaneously hypertensive rats: role of tonic vasoactive factor availability. Clinical science.

[29] Stucchi, P. *et al*. Leptin resistance develops spontaneously in mice during adult life in a tissue-specific manner. Consequences for hepatic steatosis. *Biochimie* (2011). 10.1016/j.biochi.2011.06.020 21740952

[30] Badin PM (2013). High-fat diet-mediated lipotoxicity and insulin resistance is related to impaired lipase expression in mouse skeletal muscle. Endocrinology.

[31] Wang H (2012). Obesity-induced endothelial dysfunction is prevented by deficiency of P-selectin glycoprotein ligand-1. Diabetes.

[32] Garcia-Prieto CF (2015). High-fat diet induces endothelial dysfunction through a down-regulation of the endothelial AMPK-PI3K-Akt-eNOS pathway. Molecular nutrition & food research.

[33] Ma, L. *et al*. Perivascular fat-mediated vascular dysfunction and remodeling through the AMPK/mTOR pathway in high-fat diet-induced obese rats. *Hypertens Res* **33**, 446-453, doi:hr201011 [pii]10.1038/hr.2010.11 (2010). 10.1038/hr.2010.11 20186150

[34] Grassi G (2010). Structural and functional alterations of subcutaneous small resistance arteries in severe human obesity. Obesity.

[35] Stapleton PA, James ME, Goodwill AG, Frisbee JC (2008). Obesity and vascular dysfunction. Pathophysiology: the official journal of the International Society for Pathophysiology / ISP.

[36] Khoo NK (2010). Dietary flavonoid quercetin stimulates vasorelaxation in aortic vessels. Free radical biology & medicine.

[37] Ramirez-Sanchez I, Maya L, Ceballos G, Villarreal F (2011). (-)-Epicatechin induces calcium and translocation independent eNOS activation in arterial endothelial cells. American journal of physiology. Cell physiology.

[38] Brull V (2017). Acute intake of quercetin from onion skin extract does not influence postprandial blood pressure and endothelial function in overweight-to-obese adults with hypertension: a randomized, double-blind, placebo-controlled, crossover trial. European journal of nutrition.

[39] Jimenez R (1999). Involvement of thromboxane A2 in the endothelium-dependent contractions induced by myricetin in rat isolated aorta. British journal of pharmacology.

[40] Herrera MD, Zarzuelo A, Jimenez J, Marhuenda E, Duarte J (1996). Effects of flavonoids on rat aortic smooth muscle contractility: structure-activity relationships. General pharmacology.

[41] Blanco-Rivero J (2011). Rosuvastatin restored adrenergic and nitrergic function in mesenteric arteries from obese rats. British journal of pharmacology.

[42] Liu IM, Tzeng TF, Liou SS, Lan TW (2007). Myricetin, a naturally occurring flavonol, ameliorates insulin resistance induced by a high-fructose diet in rats. Life sciences.

