# Supplementary material for: Beneficial effects of murtilla extract and madecassic acid on insulin sensitivity and endothelial function in a model of diet-induced obesity
Source: Sci Rep. 2019 Jan 24;9:599. doi: 10.1038/s41598-018-36555-1 (PMC6345770; doi:10.1038/s41598-018-36555-1)
Supplement: Supplementary file 1 — Supplementary Figures [file 41598_2018_36555_MOESM1_ESM.pdf]

SUPPLEMENTARY INFORMATION

Beneficial effects of murtilla extract and madecassic acid on insulin sensitivity and endothelial function in a model of diet-induced obesity

Jorge Arancibia-Radich[1], Raquel González-Blázquez[2], Martín Alcalá[3], Miriam Martín-Ramos[4,5], Marta Viana[3], Silvia Arribas[6], Carla Delporte[1], María S. Fernández-Alfonso[4,5], Beatriz Somoza[2]* and Marta Gil-Ortega[2]*

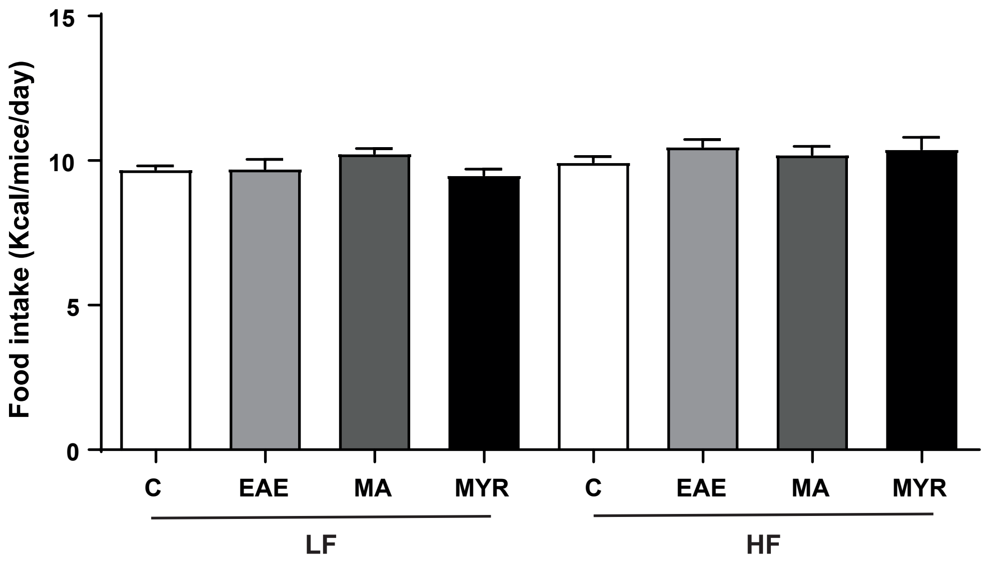

15
10
5
0
Food intake (Kcal/mice/day)
C
EAE
MA
MYR
C
EAE
MA
MYR
LF
HF

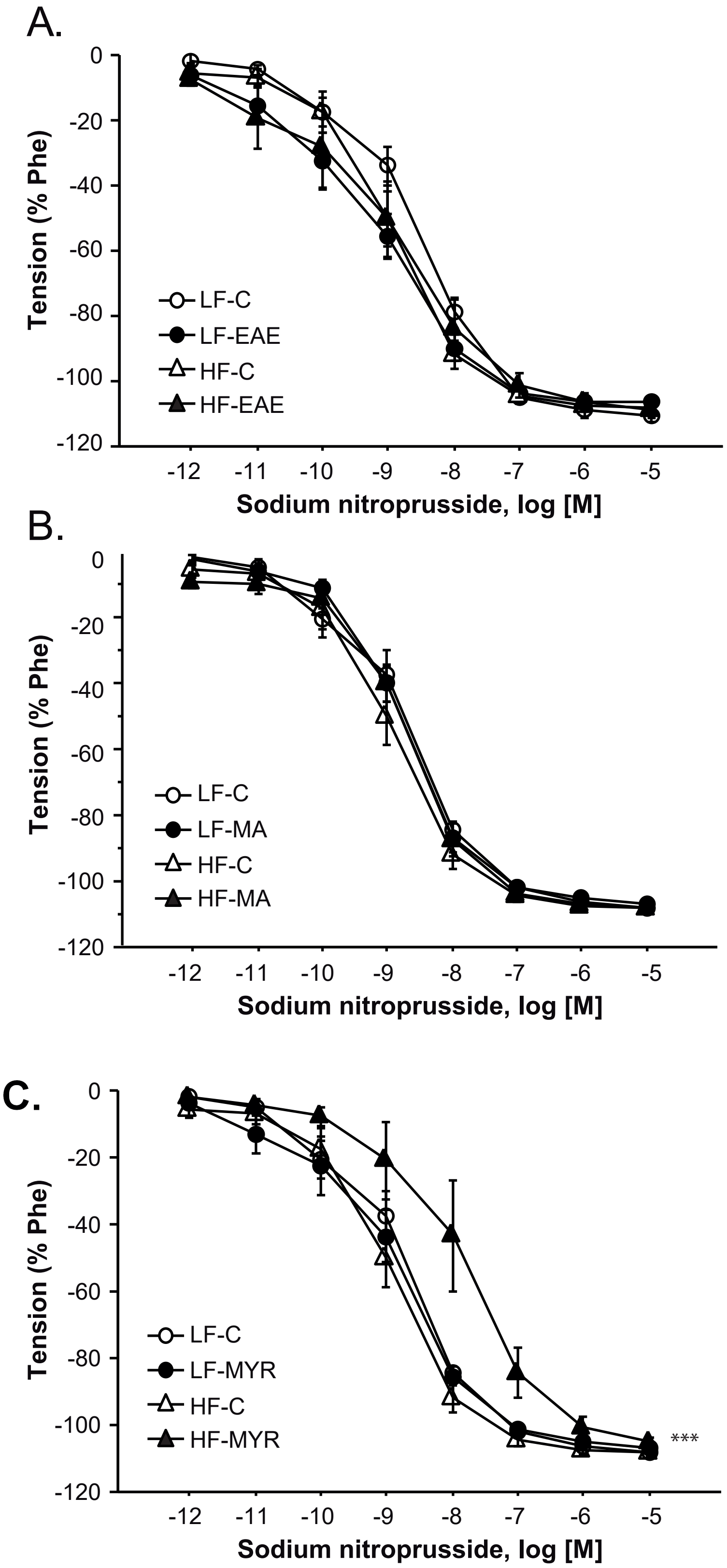
A.
Tension (% Phe)
0
-20
-40
-60
-80
-100
-120
LF-C
LF-EAE
HF-C
HF-EAE
-12
-11
-10
-9
-8
-7
-6
-5
Sodium nitroprusside, log [M]
B.
Tension (% Phe)
0
-20
-40
-60
-80
-100
-120
LF-C
LF-MA
HF-C
HF-MA
-12
-11
-10
-9
-8
-7
-6
-5
Sodium nitroprusside, log [M]
C.
Tension (% Phe)
0
-20
-40
-60
-80
-100
-120
LF-C
LF-MYR
HF-C
HF-MYR
***
-12
-11
-10
-9
-8
-7
-6
-5
Sodium nitroprusside, log [M]

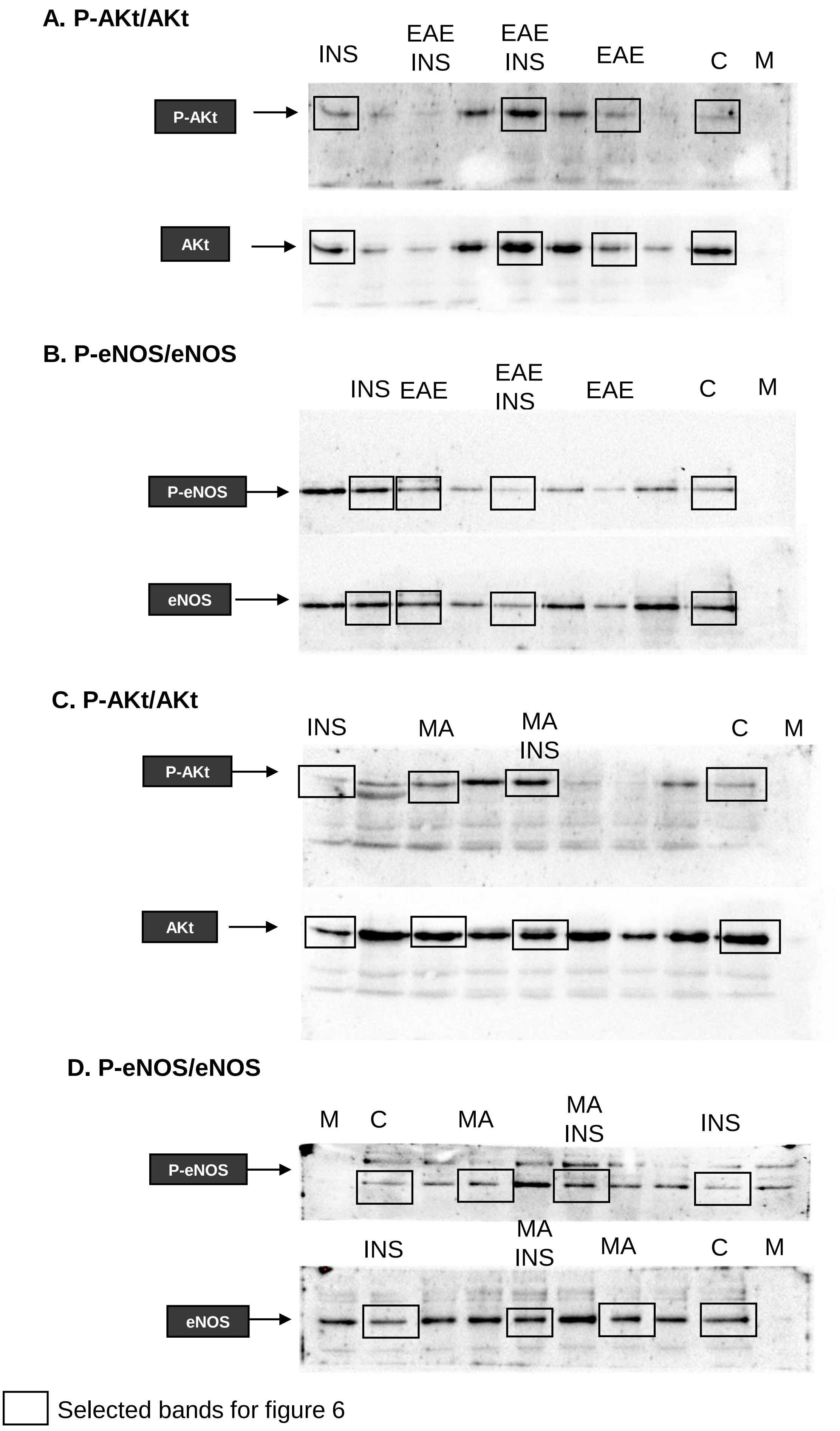
A. P-AKt/AKt
INS
EAE INS
EAE INS
EAE
C
M
P-AKt
AKt
B. P-eNOS/eNOS
INS
EAE
EAE INS
EAE
C
M
P-eNOS
eNOS
C. P-AKt/AKt
INS
MA
MA INS
C
M
P-AKt
AKt
D. P-eNOS/eNOS
M
C
MA
MA INS
INS
P-eNOS
INS
MA INS
MA
C
M
eNOS
Selected bands for figure 6

SUPPLEMENTARY FIGURE LEGENDS

Supplementary Figure 1

Diagram bars show food intake in all experimental groups. Data are expressed in Kcal/mice/day and as mean ± SEM of 5 determinations per group.

Supplementary Figure 2

Cumulative concentration-response curves to sodium nitroprusside (10^{-12}-10^{-5}M) in aortic segments from LF and HF mice treated or not with EAE (A), MA (B), or MYR (C). Data are expressed as mean ± SEM of 5 determinations per group. ***$p<0.001$ compared with the corresponding control group.

Supplementary Figure 3

Full-length blots selected for Figure 6, that constitute the most representative immunoblots of p-Akt/Akt and p-eNOS/eNOS expression.
